# Predictive Role of Population Density and Use of Public Transport for Major Outcomes of SARS-CoV-2 Infection in the Italian Population: An Ecological Study

**DOI:** 10.34172/jrhs.2021.46

**Published:** 2021-04-12

**Authors:** Alfonso Ilardi, Sergio Chieffi, Ciro Rosario Ilardi

**Affiliations:** ^1^Department of Internal Medicine, A.O.R.N. "Antonio Cardarelli Hospital", Naples, Italy; ^2^Department of Experimental Medicine, University of Campania "Luigi Vanvitelli", Naples, Italy; ^3^Department of Psychology, University of Campania "Luigi Vanvitelli", Caserta, Italy

**Keywords:** Air Pollution, COVID-19, Ecological Study, Population Density, Public Transport

## Abstract

**Background:** This study aimed at assessing how population density (PD), aging index (AI), use of public transport (URPT), and PM_10_ concentration (PI) modulated the trajectory of the main COVID-19 pandemic outcomes in Italy, also in the recrudescence phase of the epidemic.

**Study design:** Ecological study.

**Methods:** For each region, we recovered data about cases, deaths, and case fatality rate (CFR) recorded since both the beginning of the epidemic and September 1, 2020. Data about total hospitalizations were included as well.

**Results:** PD correlated with, and was the best predictor of, total and partial cases, total and partial deaths, and total hospitalizations. Moreover, URPT correlated with, and was the best predictor of, total CFR. Besides, PI correlated significantly with total and partial cases, total and partial deaths, and total hospitalizations.

**Conclusions:** PD explains COVID-19 morbidity, mortality, and severity while URPT is the best predictor of disease lethality. These findings should be interpreted with caution due to the ecological fallacy.

## Introduction

Respiratory infections, such as Coronavirus Disease 2019 (COVID-19) spread through droplets (5-10 μm) and aerosols (smaller than 5 μm) exhaled by infected individuals during quiet breathing, simple conversation, or through coughing and sneezing^1^. Hence, there is a need to comply with the recommendations provided by health authorities on social distancing (physical distancing of one meter or more) and the use of personal protective equipment. These measures, together with regular hand washing, disinfection of surfaces, and full personal hygiene, can be valuable contributions to counteract the spread of contagion^2,3^.

 After an initial decrease in cases and admissions to the intensive care unit, the contagion curve increased again with the approach of autumn. Consequently, in many European countries, including Italy, the political authorities imposed new restrictions; however, some matters remained controversial.

The increase in tourist flows, which generally occurs between July and August, may have contributed to the second wave of the pandemic. However, this hypothesis is more applicable to the countries of the northern hemisphere, rather than the countries of the southern hemisphere where the summer season begins on December 21^st^ and ends on March 20^th^.

Recent studies have suggested that population density (PD) is one of the key factors favoring transmissibility^4,5^. The use of public transport has also been reported as a possible factor increasing the spread of contagion^6^. The number of public transport users is higher in the most densely populated territories, and the restricted environments in which the transport occurs may not allow social distancing. Although the disease primarily affects older and multimorbid adults, the results of previous investigations have indicated that the aging index (AI) does not affect COVID-19 morbidity, mortality, and lethality ^4^. There are also non-demographic variables that could moderate the impact of the COVID-19 outbreak. For instance, it has been hypothesized that PM_10 _concentrations exceeding 0.05 mg/m^3^ may result in accelerated dissemination of the virus ^7^.

 In light of issues such as those raised above, the present ecological study aimed at estimating the contribution of PD, AI, public transport use, and PM10 concentration in modulating the trajectory of the main COVID-19 pandemic outcomes in Italy, also in the recrudescence phase of the epidemic.

## Methods

To detect any relationships between the available demographic/epidemiological data and the impact of COVID-19 pandemic on the Italian population ^8-13^, we characterized each of the 20 Italian regions based on the PD (i.e., number of inhabitants per km^2^), AI (i.e., the number of elders per 100 persons younger than 15 years old; a value higher than 100 indicates a higher number of older subjects than younger ones), utilization rate of public transport users (URPT), annual average of PM_10_ daily mean concentration (pollution index [PI]), total number of positive cases, total number of hospitalized patients (i.e., ordinary hospitalization and intensive care), total number of deaths, and case fatality rate (CFR) (i.e., the proportion of deceased patients among the total number of positive cases).

The data about the total number of cases, deaths, hospitalizations, and the total CFR, were collected from the beginning of the epidemic until November 4, 2020. Our archival research also included the partial estimate of cases, deaths, and CFR calculated from September 1, 2020, until November 4, 2020. These estimates will be referred to as partial cases, partial deaths, and partial CFR, respectively (Table 1). Data about PD, AI, and URPT were provided by the Italian National Institute of Statistics^10-12^ while those about IP were collected from the Global Health Observatory (World Health Organization)^13^.

**Table 1:** Descriptive statistics for each region (Data updated on November 4, 2020)

**Region**	**PD** **(Inhab /Km**^2^**)**	**AI (%)**	**URPT (%)** ^a^	**PI** ^b^	**Total cases**	**Total deaths**	**Total hospitalizations** ^c^	**Partial cases** ^d^	**Partial deaths** ^d^	**CFR (%)**	**Partial** **CFR (%)** ^d^
Abruzzo	120.6	197.7	55.5	24.3	12,543	568	463	8,787	96	4.53	1.09
Basilicata	55.3	200.5	50.4	18.7	2,788	54	104	2,266	26	1.94	1.15
Calabria	126.4	169	46.3	22.9	6,092	125	223	4,602	27	2.05	0.59
Campania	423.2	134.7	49.9	31.1	69,613	739	1,744	62,505	294	1.06	0.47
Emilia Romagna	199.0	186.4	62.2	24.8	62,914	4,699	1,715	31,031	236	7.47	0.76
Friuli Venezia Giulia	152.7	223.1	58.6	21.9	12,264	414	255	8,495	66	3.38	0.78
Lazio	340.4	167.7	57.7	25.3	55,273	1,309	2,534	44,082	431	2.37	0.98
Liguria	284.9	260.7	53.2	20.7	32,117	1,834	1,273	21,166	263	5.71	1.24
Lombardia	423.4	169.7	60.3	29.5	224,191	17,848	5,525	124,116	983	7.96	0.79
Marche	161.5	202.3	58.4	23.9	16,261	1,032	452	9,021	45	6.35	0.50
Molise	67.8	225.5	50.9	18.9	2,016	41	35	1,491	18	2.03	1.21
Piemonte	171.0	211.3	56	26.3	81,409	4,481	3,758	48,528	335	5.50	0.69
Puglia	205.1	175.4	49.8	23.2	22,085	763	861	16,645	207	3.45	1.24
Sardegna	67.7	221.6	49.9	22.4	10,640	241	389	8,447	107	2.27	1.27
Sicilia	192.3	159	47	21.7	26,080	569	1,253	21,763	283	2.18	1.30
Toscana	161.9	209.8	59.6	22.7	52,815	1,461	1,516	40,957	304	2.77	0.74
Trentino-Alto Adige	79.0	142	62.8	18.1	19,969	773	564	11,938	76	3.87	0.64
Umbria	104.0	210.5	55.2	22.2	12,056	154	356	10,263	74	1.28	0.72
Valle d’Aosta	38.5	188.2	61.6	21.4	3,720	181	163	2,479	35	4.87	1.41
Veneto	267.5	178.2	61.7	27.6	65,531	2,478	1,225	42,602	358	3.78	0.84

PD: population density; AI: aging index; URPT: utilization rate of public transport; PI: pollution index a^a^Utilization rate of public transport users in 2019 ^b^ Annual average of PM_10_ daily mean concentration (mg/m^3^) during 2013-2016 ^c^ Ordinary hospitalization and intensive care ^d^ Partial estimate of cases, deaths, and CFR since September 1, 2020

In particular, data about URPT were acquired through a mixed approach (i.e., computer-assisted web interviews, computer-assisted personal interviews, and paper-and-pencil interviews) involving approximately 25,000 families distributed across about 800 Italian municipalities of different demographic sizes^12^. Conversely, the data about PI were regularly acquired and processed through monitors located within the main Italian metropolitan areas ^13^. Variables concerning the COVID-19 outcomes were finally extracted from the historical data provided by the Italian Ministry of Health and National Health Institute ^8,9^.

 Statistical analyses were performed by means IBM SPSS Statistics for Windows (version 26). Bivariate correlation analysis was used for exploratory purposes. Cohen’s conventions (weak: <0.30; moderate: 0.30–0.50; strong: >0.50) were employed to interpret effect size. Subsequently, stepwise regression analyses were performed to study the relationship of sociodemographic (i.e., PD, AI, URPT) and environmental (i.e., PI) variables with the morbidity, mortality, severity, and lethality of COVID-19.

 We ran seven stepwise regression models entering PD, AI, URPT, and PI as independent variables, and total cases, total hospitalized patients, total deaths, total CFR, partial cases, partial deaths, and partial CFR as separate dependent ones. In SPSS, the stepwise regression is a statistical data-driven regression technique combining forward and backward regression methods. The analysis started with no independent variables in the model.

At each step, the predictor explaining the largest amount of variance was added to the model. Variables entered in the model were systematically re-evaluated to establish whether their unique contribution to the variance was still significant after the addition of other predictors. Candidate predictors were dropped if they were no longer significant^14^. For all analyses, the significance threshold was set at α≤0.05 and adjusted according to Bonferroni’s correction method.

## Results

About half of the variables under examination (i.e., total cases, total hospitalized patients, total deaths, partial cases, and partial deaths) did not satisfy the assumption of normality based on the examination of skewness and kurtosis values and results of the Shapiro-Wilk test. Therefore, non-parametric Spearman’s correlations (r_rho_) were performed. Table 2 summarizes the results of correlation analyses.

**Table 2:** Correlation matrix

**Variables**	**PD**	**AI**	**URPT**	**PI**	**Total** **cases**	**Total** **deaths**	**Total ** **hospitalizations**	**Partial** **cases**	**Partial** **deaths**	**CFR**	**Partial** ** CFR**
Population density	-	–0.36	0.05	0.70^*^	0.87^*^	0.75^*^	0.84^*^	0.87^*^	0.81^*^	0.27	–0.21
Aging index	–0.36	-	–0.01	–0.34	–0.35	–0.17	–0.35	–0.40	–0.31	0.09	0.21
Utilization rate of public transport	0.05	–0.01	-	0.11	0.32	0.53	0.23	0.26	0.23	0.62^*^	–0.21
Pollution index	0.70^*^	–0.34	0.11	-	0.73^*^	0.58^*^	0.68^*^	0.72^*^	0.67^*^	0.24	–0.39

Adjusted alpha (α/7)=0.007 *P ≤ 0.007

The PD showed a strong association with total cases (r_rho_=0.87, *P*<0.001), total hospitalized patients (r_rho_=0.84, P<0.001), and total deaths (r_rho_=0.75, *P*<0.001). Furthermore, PD had a strong correlation with partial cases (r_rho_=0.87, *P*<0.001) and deaths (r_rho_=0.81, *P*<0.001), while it did not correlate with total or partial CFR. Moreover, AI did not correlate with any of the variables under examination, while URPT showed a strong correlation with total CFR (r_rho_=0.62, *P*=0.003). Finally, PI had a significant and strong association with total (r_rho_=0.73, *P*<0.001) and partial cases (r_rho_=0.72, P<0.001), total (r_rho_=0.58, *P*=0.007) and partial deaths (r_rho_=0.67, *P*=0.001), and total hospitalized patients (r_rho_=0.68, *P*=0.001). Nevertheless, no correlation was found between PI and total or partial CFR.

As shown in Table 2, PD and PI were strongly interrelated (r_rho_=0.70, *P*<0.001). The PD and PI as well as AI and URPT entered seven regression models as candidate predictors of each outcome variable. Therefore, the finding of a bivariate relationship between these two variables suggested further examination of the presence of multicollinearity. In the case of correlation among predictors in a regression analysis, the variance of regression coefficients can be inflated. Therefore, we calculated the variance inflation factor (VIF) for each predictor to determine whether and how much the variance of regression coefficients was inflated.

The following general rules were applied to interpret the VIF values: VIF=1, no collinearity; VIF=1 to 5, moderate collinearity; VIF>5, high collinearity^15^. The variances of PD and PI coefficients were inflated by factors of 2.71 and 2.86, respectively. Furthermore, the tolerance values (i.e., the amount of variability not explained by the other independent variables) were higher than 0.10 (PD=0.37, PI=0.36) which indicates the absence of statistically significant multicollinearity ^15^.

Overall, these results suggest the presence of moderate multicollinearity but not severe enough to affect the interpretation of regression models or warrant further corrective measures. Besides excluding multicollinearity, other assumptions of linear regression were satisfied: 1) both independent and dependent variables were quantitative, ii) there was a linear relationship between outcome and independent variables, and iii) the standardized residuals showed no drastic deviation from normality based on inspection of both P-P plots and histograms. Table 3 tabulates the results of regression analyses.

It was found that PD was the best predictor of the main pandemic outcomes. Particularly, the variances of total and partial cases, total and partial deaths, and total hospitalizations were explained only by increasing PD (total cases: R^2^=0.55, B=326.93, *P*<0.001; partial cases: R^2^=0.68, B=211.36, *P*<0.001; total deaths: R^2^=0.32, B=19.65, *P*=0.009; partial deaths: R^2^=0.64, B=1.57, *P*<0.001; total hospitalizations: R^2^=0.51, B=8.65, *P*<0.001). The variance of total CFR was explained by URPT; accordingly, a higher URPT predicted an increase in total CFR (R^2^=0.35, B=0.22, *P*=0.006). However, none of the candidate predictors sufficiently explained the variance of partial CFR.

## Discussion

Based on the results of the present study, it is confirmed that PD increases the likelihood of interpersonal contacts and hence viral transmission. In line with previous studies, PD was found to be associated with the number of cases and deaths^4^. Moreover, PD showed a positive linear association with the total number of hospitalizations. This result suggests that the likelihood of developing the “severe form” of the COVID-19 that requires hospitalization is higher in the most populated regions of Italy. Furthermore, PD was strongly associated with the number of cases and deaths recorded during the second wave of the pandemic.

These findings, together with the confirmatory results of regression analysis, prove that PD is still the best demographic predictor of the spread of contagion and mortality of SARS-CoV-2 in the Italian population, also in the recrudescence phase of the epidemic. Unlike the findings of our previous study ^4^, no positive relationship was found between PD and CFR. In this respect, some important considerations need to be discussed.

 On the one hand, in our previous study, the statistical adjustment for multiple comparisons was not applied to avoid being too conservative. On the other hand, it should also be noted that, compared to the first months of the epidemic, a more widespread use of nasopharyngeal swabs for the assessment of cases, even asymptomatic ones, has involved increasingly broader population groups. Early detection of cases and their isolation might have contributed to the limitation of the viral circulation within the most vulnerable populations which, in turn, has led to lower CFR and the loss of correlation.

According to the results of regression analysis, URPT was the best predictor of total CFR. In other words, sharing the confined environment of a public transport vehicle or crowded public transport stations might increase the lethality of SARS-CoV-2. On average, people using public transports are younger (students and workers), and the infection tends to progress in an apparently asymptomatic manner or with few symptoms in younger subjects ^16^. Therefore, this may have favored the circulation of asymptomatic/paucisymptomatic cases, and consequently the transmission of coronavirus to the most vulnerable populations including older adults and multimorbid patients.

**Table 3:** Predictors of the main COVID-19 pandemic outcomes

**Variables**	**B**	**t**	**P**	**F**	**R** ^2^
Total cases				F (1,18)=22.215, *P*<0.001	0.55
Population density	326.93	4.713	0.001		
Aging index	–0.01	–0.06	0.950		
Utilization rate of public transport	0.28	1.910	0.073		
Pollution index	0.31	1.219	0.240		
Total hospitalized patients				F (1,18)=19.086, *P*<0.001	0.51
Population density	8.65	4.369	0.001		
Aging index	0.04	0.21	0.836		
Utilization rate of public transport	0.21	1.324	0.203		
Pollution index	0.27	0.99	0.338		
Total deaths				F (1,18)=8.518, * P*=0.009	0.32
Population density	19.65	2.919	0.009		
Aging index	0.06	0.30	0.765		
Utilization rate of public transport	0.31	1.667	0.114		
Pollution index	0.22	0.675	0.509		
Total case fatality rate				F (1,18)=9.581, *P*=0.006	0.35
Population density	0.19	0.997	0.333		
Aging index	0.16	0.815	0.427		
Utilization rate of public transport	0.22	3.095	0.006		
Pollution index	0.17	0.874	0.394		
Partial cases				F (1,18)=38.700, *P*<0.001	0.68
Population density	211.36	6.221	0.001		
Aging index	–0.06	–0.395	0.698		
Utilization rate of public transport	0.21	1.669	0.113		
Pollution index	0.32	1.514	0.148		
Partial deaths				F (1,18)=32.089, *P*<0.001	0.64
Population density	1.57	5.665	0.001		
Aging index	0.02	0.148	0.884		
Utilization rate of public transport	0.19	1.417	0.175		
Pollution index	0.09	0.380	0.709		
Partial case fatality rate				F (1,18)=4.425, *P*=0.050	0.20
Population density	0.13	0.353	0.728		
Aging index	0.18	0.790	0.442		
Utilization rate of public transport	–0.22	–1.028	0.318		
Pollution index	–0.04	–2.104	0.050		

Adjusted alpha (α/4)=0.01

As previously mentioned, no association was found between AI and the main pandemic outcomes. Generally speaking, the females are the least affected, and the mean survival rate is higher in elderly multimorbid females with COVID-19 ^17^. Since the infection affects males more than females ^18,19^, the higher prevalence of females in the older age groups of the Italian population may have blunted the association of AI with morbidity, mortality, disease severity, and lethality of SARS-CoV-2.

It is widely acknowledged that biological and chemical components of air pollution can have a negative impact on human health. Air pollution represents a well-known cause of prolonged inflammation as a result of an over-expression of inflammatory cytokines, even in healthy subjects ^20^. Based on previous research, there is a positive association between the time of exposure to particulate matter, prior to the pandemic period, and the increase in vulnerability to SARS-CoV-2 ^21^. Furthermore, air pollution appears to increase the risk of mortality from COVID-19 ^22^. In line with this evidence, we found that PI has a significant correlation with morbidity, mortality, and total number of hospitalizations; however, no correlation was observed between PI and CFR. It should be noted that the greatest exposure to PM_10_ is likely prevalent in younger subjects who use public transport daily for study or work needs.

Ecological fallacy is the main limitation of the present study. Ecological fallacy is a bias affecting the interpretation of results since a relationship observed among variables on an aggregate level does not necessarily represent with confidence the association existing at an individual level ^23^. For instance, from a statistical standpoint, a correlation tends to be stronger when an association is assessed at an aggregate level rather than an individual level. Still, details about individual profiles may be missed when analyses are conducted on aggregate data. Consequently, the findings of the present study should be interpreted with caution.

## Conclusion

 The current pandemic has brought to the attention of political and health authorities several issues that directly affect the daily lives of citizens. These issues extend from travel patterns within the cities of residence to the frequency of schools. Epidemiological studies involving demographic and environmental variables are needed to help policy-makers and administrative authorities to make informed and targeted decisions that can substantially change daily life.

 In the present study, it was confirmed that PD plays a striking role in exacerbating the spread of contagion, and its relationship with disease severity and mortality. Furthermore, a strong association was found between URPT and COVID-19 lethality. It was hypothesized that the use of public transport by younger individuals, who are more likely to be asymptomatic, may promote the circulation of the virus and, consequently, its spread to more vulnerable individuals.

 The limitation of ecological studies, such as this one, is that they provide trend information only on aggregated data. Nevertheless, the present study, which “photographs” the impact of some relevant aspects of the current pandemic in the Italian context, could serve as a guide for political and administrative management, and as a warning for future research.

## Conflict of interest

 The authors have no conflicts of interest to declare.

## Funding

 No funding to declare.

## Highlights

- Population density, frequent use of public transports, and larger PM_10_ concentrations may modulate the trajectory of the main COVID-19 pandemic outcomes.
- Population density is the best predictor of morbidity, mortality, and disease severity.
- Higher use of public transports explains the fatality of COVID-19.
